# Optimization-Based Sensor Fusion of GNSS and IMU Using a Moving Horizon Approach

**DOI:** 10.3390/s17051159

**Published:** 2017-05-19

**Authors:** Fabian Girrbach, Jeroen D. Hol, Giovanni Bellusci, Moritz Diehl

**Affiliations:** 1 Xsens Technologies B.V., Enschede 7521 PR, The Netherlands; jeroen.hol@xsens.com (J.D.H.); giovanni.bellusci@xsens.com (G.B.); 2 Department of Microsystems Engineering (IMTEK), University of Freiburg, 79110 Freiburg, Germany; moritz.diehl@imtek.uni-freiburg.de

**Keywords:** multi-sensor fusion, state estimation, moving horizon estimation, nonlinear optimization, inertial navigation

## Abstract

The rise of autonomous systems operating close to humans imposes new challenges in terms of robustness and precision on the estimation and control algorithms. Approaches based on nonlinear optimization, such as moving horizon estimation, have been shown to improve the accuracy of the estimated solution compared to traditional filter techniques. This paper introduces an optimization-based framework for multi-sensor fusion following a moving horizon scheme. The framework is applied to the often occurring estimation problem of motion tracking by fusing measurements of a global navigation satellite system receiver and an inertial measurement unit. The resulting algorithm is used to estimate position, velocity, and orientation of a maneuvering airplane and is evaluated against an accurate reference trajectory. A detailed study of the influence of the horizon length on the quality of the solution is presented and evaluated against filter-like and batch solutions of the problem. The versatile configuration possibilities of the framework are finally used to analyze the estimated solutions at different evaluation times exposing a nearly linear behavior of the sensor fusion problem.

## 1. Introduction

**Figure 1:**
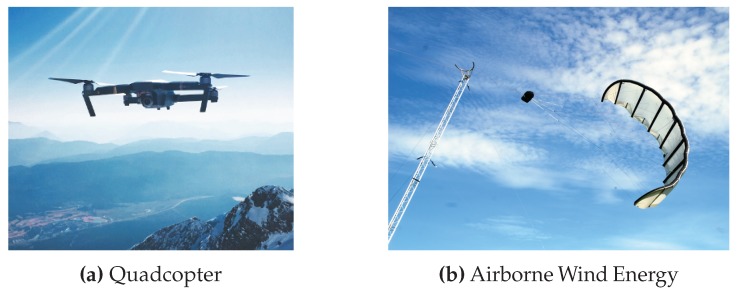
Examples for autonomous airborne applications: (**a**) shows a flying drone (available at https://unsplash.com/photos/ZlkRrzJl20Q, published under CC0, photo by imKirk); (**b**) shows a prototype of an airborne wind energy system developed at TU Delft (http://www.kitepower.eu). Courtesy of Roland Schmehl, TU Delft.

During the last few years, fully autonomous systems have been a highly active research field, which pushed product development towards the commercialization of such systems. For applications such as autonomous driving and *unmanned aerial vehicles* (uavs) more semi-autonomous features become available every day. Besides the future of self-driving cars, autonomous drones will soon take over tasks in transportation, agriculture, maintenance, surveillance or energy generation. The first prototypes have already proven to be feasible and have successfully delivered small sized goods, simplified inspection processes in rough terrain or harvested wind energy at previously unreachable altitudes. These recent developments allow the prediction of an increasing number of uavs applications (see Figure 1), which will result in a more crowded airspace, representing a paradigm shift in comparison to traditional airborne applications where the airspace is heavily secured and supervised.

Uavs operating in a more occupied airspace close to humans require increased robustness to avoid fatal incidents, which translates to a strict set of requirements imposing new challenges for the estimation and control algorithms. *Moving horizon estimation* (mhe) and *model predictive control* (mpc) are promising strategies that use numerical optimization methods over a window of data to increase stability and accuracy of the system’s motion. Mpc has been successfully applied to challenging control problems with nonlinear dynamics and difficult environment conditions [1,2,3]. The counterpart to the optimal control theory for state estimation is represented by mhe which has already been used for decades to estimate the state of nonlinear chemical processes [4] or more recently, to increase fault tolerance of relative navigation problems [5]. The computational burden of solving an optimization problem has become less restrictive due to advances in computer technology and the development of tailored optimization algorithms [6,7] that nowadays allo the usage of efficient optimization-based methods on embedded systems. These recent developments make these strategies attractive for a broad range of applications including autonomous systems.

To estimate the motion of a system exposing a high degree of freedom with high accuracy, it often becomes necessary to fuse information from different sensors. Sensor fusion is a well known strategy to reduce the impact of measurement errors on the state estimate [8] and estimate not directly observable system states. One traditional way to solve multi-sensor fusion problems for time critical applications is the famous *Kalman filter* (kf) algorithm [9] and its derivatives for nonlinear systems *extended Kalman filter* (ekf) and *unscented Kalman filter* (ukf). To achieve high estimation accuracy, kf-based algorithms require, in practice, a procedure to tune the filter parameters, which is, for complex systems, non-trivial yet crucial. To overcome this problem, adaptive methods have been proposed [10,11]; however, filter stability can become an issue under certain conditions. Optimization-based approaches allow for a more elegant formulation respecting system constraints [12]. Typical Newton-type optimization algorithms use an iterative approach and are therefore known to better capture the nonlinearity of the problem yielding a more accurate and robust solution.

In this paper, we present an optimization-based sensor fusion framework for state estimation following an mhe approach, extending [13]. The framework is applied to the well-known sensor fusion problem for inertial navigation of a *global navigation satellite system* (gnss) receiver measuring position and an *inertial measurement unit* (imu) measuring linear acceleration and angular velocity. The measurement data is used to estimate the 3D-pose and velocity of a maneuvering object, such as an aircraft or satellite. There are several methods to integrate the inertial data and gnss, such as loosely coupled and tightly coupled integration. The integration is typically achieved by adapting a nonlinear kf [14,15,16]. By preprocessing the nonlinearities of the problem, linear kfs represent a further option for the sensor fusion problem [17].

The sensor fusion problem discussed in this paper arises from the use of consumer-grade imus for which sensor errors introduce a drift that needs to be compensated for by using different measurement updates such as those given by vision based sensors [18] or gnss receivers. The resulting sensor fusion problem for inertial navigation has been addressed using traditional and advanced approaches for Kalman filtering [17,19]. The observability of sensor parameters, such as biases of the imu, is strongly coupled to the motions of the system. Therefore, a unique solution to the estimation problem cannot be guaranteed [20]. By considering a time window of measurement data, the estimated solution is suggested to achieve improved accuracy and robustness [21]. Several mhe formulations for sensor fusion in the context of inertial navigation have been published in the recent past and have been shown to outperform traditional ekf approaches for the integration of gnss and imu [22,23] and online-identification of imu parameters [24]. Despite the different sets of sensors, models and optimization methods, all authors present an accuracy improvement for a specific horizon length compared to Kalman filtering. This paper contributes to the existing research by presenting a general sensor fusion framework capable of analyzing moving horizon estimators over the spectrum of filter-like configurations to batch processing. Furthermore, the estimated results are evaluated against an accurate reference of a maneuvering aircraft that was obtained using high-grade sensors. A detailed study of the influence of the horizon length on the quality of the solution is given and critically analyzed.

This paper is organized as follows. First, in Section 2, we describe the relevant system and sensor models to address the sensor fusion problem by the definition of an equality-constrained optimization problem, which components are explained in detail. In Section 3, the presented approach is used to estimate the trajectory of an aircraft, and the results are compared against an accurate reference trajectory. Finally, the conclusions of this work are presented in Section 4.

## 2. Methods

Section 2 introduces the necessary coordinate frames to define the system and sensor models, which will finally be used to derive the optimization problem of the mhe state estimator.

### 2.1. Coordinate Frames

**Figure 2:**
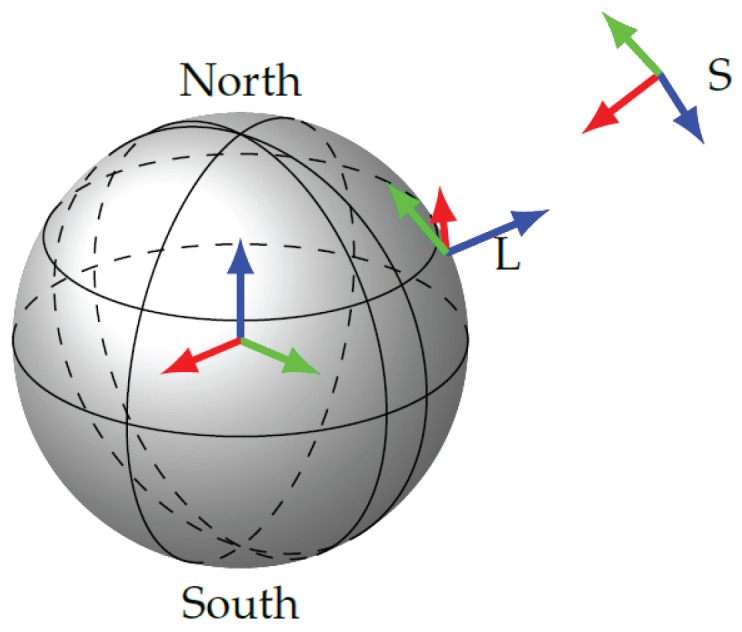
The relevant navigation frames for the fusion of gnss and imu. The figure shows the fixed frames (ecef and *L*) and the free moving sensor frame *S*.

The sensor fusion problem contains measured and estimated quantities expressed in several coordinate frames (see Figure 2). The position measurements are obtained by the gnss sensor in the *earth-centered, earth-fixed* (ecef) frame and often expressed in *latitude, longitude, altitude* (lla). The measurements are transformed to a locally-fixed and non-moving frame *L* following the *east, north, up* (enu) convention with its origin located at a reference location. Since the transformation between global and local frame is constant over time, the measurements are converted to the local frame in a preprocessing step. The measurements of the imu are obtained in the sensor coordinate frame *S*, which is moving with respect to the local frame *L*. Throughout this document, the notation $\cdot^{L}$ or $\cdot^{S}$ is adopted to indicate measured or estimated variables according to the local or sensor frame, respectively.

### 2.2. System Model

A generic piecewise constant linear acceleration and angular velocity model are used to model translational and rotational dynamics of the moving system. These models are widely used in the target tracking community and can be reviewed in detail in, e.g., the survey of [25]. The *ordinary differential equations* (odes) of the navigational states are defined by

(1a) $$\begin{array}{ccc} {\dot{p}}^{L}\left(t\right) & = & v^{L}\left(t\right), \end{array}$$

(1b) $$\begin{array}{ccc} {\dot{v}}^{L}\left(t\right) & = & a^{L}\left(t\right), \end{array}$$

(1c) $$\begin{array}{ccc} {\dot{q}}^{LS}\left(t\right) & = & \frac{1}{2}q^{LS}\left(t\right)⊙\omega_{LS}^{S}\left(t\right), \end{array}$$

where the position $p^{L}\left(t\right)\in R^{3}$ and velocity $v^{L}\left(t\right)\in R^{3}$ are obtained by integration of the acceleration $a^{L}\left(t\right)\in R^{3}$. The angular velocity $\omega_{LS}^{S}\in R^{3}$ from the *S*-frame to the *L*-frame expressed in the *S*-frame drives the ode of the orientation, which is parametrized by a unit quaternion $q^{LS}\left(t\right)\in Q_{1}=\{R^{4}{:∥q∥}_{2}=1\}$ describing the orientation between the *S* and *L*-frame. The ⊙ operator is introduced

(2) $$q⊙r\left[-q_{v}\cdot r,q_{0}r+q_{v}\times r\right]$$

for the product of a vector $r\in R^{3}$ and a quaternion $q=\left(q_{0},q_{v}\right)\in Q_{1}$ with $q_{0}\in R$ and $q_{v}\in R^{3}$ being the scalar and vector part, respectively. The ode Equations (1) of the system model lead to the definition of the concatenated vector

(3) $$x_{M}\left(t\right)=[p^{L}{\left(t\right)}^{T},v^{L}{\left(t\right)}^{T},q^{LS}{\left(t\right)}^{T}{]}^{T},$$

which contains the navigation states of the model and is therefore denoted by the index *M*. The navigational states are driven by the piecewise constant control inputs

(4) $$u\left(t\right)=[a^{L}{\left(t\right)}^{T},\omega_{LS}^{S}{\left(t\right)}^{T}{]}^{T}.$$

### 2.3. Sensor Models

The sensors used for this estimation problem are a single gnss receiver and an imu which are both rigidly attached to the moving system. The described sensor models relate the measured quantities to the state and control variables defined accordingly in Equations (3) and (4). Section 2.2 defines a continuous-time model; however, the sensors acquire the measurements at discrete times $t_{k}$ with a sensor-specific measurement frequency $f_{S}$. We define output functions for each sensor measurement, which evaluate the continuous variables at the sampling times $t_{k}$ and establish the relation between discrete measurements and estimated continuous state and control variables.

#### 2.3.1. Inertial Measurement Unit

An imu combines a *three-axis accelerometer and a three-axis* gyroscope in a single package. The sensors can be based on different principles that define implicitly the accuracy of the sensor. Due to the advances in *micro-electro-mechanical system* (mems) technology, the sensor modules can be produced very cost efficiently on a single silicon chip. The mems accelerometer and gyroscope are modeled in this paper using an additive bias term $\delta^{S}$ to compensate the time-varying offsets in the average signal output of the sensor [26]. These biases $\delta_{a}^{S}\in R^{3}$ and $\delta_{\omega }^{S}\in R^{3}$ are modeled as a random walk using the device specific Allan variance [27]. To include the estimation of the bias terms, we extend the state vector $x_{M}\left(t\right)$ as defined in Equation (3) to

(5) $$x\left(t\right)=[x_{M}{\left(t\right)}^{T},\delta_{a}^{S}{\left(t\right)}^{T},\delta_{\omega }^{S}{\left(t\right)}^{T}{]}^{T}=[p^{L}{\left(t\right)}^{T},v^{L}{\left(t\right)}^{T},q^{LS}{\left(t\right)}^{T},\delta_{a}^{S}{\left(t\right)}^{T},\delta_{\omega }^{S}{\left(t\right)}^{T}{]}^{T}.$$

The bias terms are modeled as constants between consecutive sampling times $t_{k}$ and $t_{k+1}$ of the sensor exposing the lowest sampling rate $f_{S}$ leading to

(6a) $$\begin{array}{cc} {\dot{\delta }}_{a}^{S}\left(t\right) & =\mathrm{const},\;t_{k}\geq t>t_{k+1}, \end{array}$$

(6b) $$\begin{array}{cc} {\dot{\delta }}_{\omega }^{S}\left(t\right) & =\mathrm{const},\;t_{k}\geq t>t_{k+1}, \end{array}$$

and the actual random walk is embedded inside the optimization problem by allowing discontinuities according to the device specific random walk process noise.

The inertial quantities acceleration $y_{a}^{S}\in R^{3}$ and angular velocity $y_{\omega }^{S}\in R^{3}$ are acquired by the imu at sampling rate $f_{\mathrm{IMU}}\gg 100\;\mathrm{Hz}$. The sampled imu data is integrated between two consecutive measurements of the slowest sensor, which is, in this specific problem, the gnss receiver, and expressed as motion increments [28]. The increments are converted to an average inertial measurement over the interval $\left[t_{k},t_{k+1}\right]$, which directly translates to the piecewise constant control inputs defined in Equation (4). The following output functions define the imu measurements in terms of state and control variables:

(7a) $$\begin{array}{cc} y_{a}^{S}\left(x,u,t_{k}\right) & =R\left({q^{LS}\left(t_{k}\right)}^{-1},a^{L}\left(t_{k}\right)-g^{L}\right)+\delta_{a}^{S}\left(t_{k}\right), \end{array}$$

(7b) $$\begin{array}{cc} y_{\omega }^{S}\left(x,u,t_{k}\right) & =\omega_{LS}^{S}\left(t_{k}\right)+\delta_{\omega }^{S}\left(t_{k}\right), \end{array}$$

where $g^{L}$ stands for the constant gravity vector in the *L* frame and R(q,r) denotes the rotation of a vector $r\in R^{3}$ by a unit quaternion $q\in Q_{1}$.

#### 2.3.2. GNSS Receiver

A gnss receiver is used to retrieve position measurements based on pseudo ranges estimated from satellite signals. The interested reader is referred to [29] for more information. A typical gnss receiver has an output frequency between 1Hz and 10Hz, at which the position measurements are made available with respect to an earth fixed coordinate frame and converted to the local *L* frame as described before in Section 2.1. The output function for position measurements at $t_{k}$ is defined by

(8) $$y_{p}^{L}\left(x,u,t_{k}\right)=p^{L}\left(t_{k}\right),$$

which relates the gnss measurements directly to the state x(t). The position accuracy is estimated and reported by the gnss receiver and strongly depends on the signal and ambient conditions.

### 2.4. Optimization Problem

To define the optimization problem for the nonlinear sensor fusion of gnss and imu, we first define the components of the cost function, the implicit integration method using direct collocation, and the necessary equality constraints.

#### 2.4.1. Measurement Residuals

The cost function of the optimal estimation problem is defined by the squared weighted sum of residuals between the estimated output variables $y_{k}$ and the measurements ${\bar{y}}_{k}$ at sampling times $t_{k}$. The cost terms for the position and inertial measurements are defined by

(9a) $$\begin{array}{cc} c_{p}\left(x,u,t_{k}\right) & =\frac{1}{2}{∥r_{p}\left(x,u,t_{k}\right)∥}_{Q_{p}^{-1}}^{2}=\frac{1}{2}{∥y_{p}^{L}\left(x,u,t_{k}\right)-{\bar{y}}_{p,k}^{L}∥}_{Q_{p,k}^{-1}}^{2}, \end{array}$$

(9b) $$\begin{array}{cc} c_{a}\left(x,u,t_{k}\right) & =\frac{1}{2}{∥r_{a}\left(x,u,t_{k}\right)∥}_{Q_{a}^{-1}}^{2}=\frac{1}{2}{∥y_{a}^{S}\left(x,u,t_{k}\right)-{\bar{y}}_{a,k}^{S}∥}_{Q_{a}^{-1}}^{2}, \end{array}$$

(9c) $$\begin{array}{cc} c_{\omega }\left(x,u,t_{k}\right) & =\frac{1}{2}{∥r_{\omega }\left(x,u,t_{k}\right)∥}_{Q_{\omega }^{-1}}^{2}=\frac{1}{2}{∥y_{\omega }^{S}\left(x,u,t_{k}\right)-{\bar{y}}_{\omega ,k}^{S}∥}_{Q_{\omega }^{-1}}^{2}, \end{array}$$

and evaluate the continuous output functions of imu (7) and gnss (8) at time $t_{k}$. The residuals are weighted according to the measurement noise variances using the diagonal matrices $Q_{\left(\cdot \right)}$.

#### 2.4.2. Direct Collocation

We use a direct collocation approach to embed the integration of the nonlinear dynamics inside the optimization problem. Direct collocation is a strategy known from the field of direct optimal control [30] that is based on the discretization of control and state trajectory, therefore lifting the problem to higher dimensional space in comparison to traditional single shooting methods [31]. By embedding the numerical integration inside the optimization problem using collocation variables, the need of an explicit call to an integrator vanishes. We decided to adopt direct collocation for the presented sensor fusion problem because of the potential to decrease the number of iterations by initializing the collocation variables well. The discretization of x(t) and u(t) at times $t_{0:N}$ leads to the discrete sets of states $X=\{x_{0}\ldots x_{N}\}$ and controls $U=\{u_{0}\ldots u_{N-1}\}$. While the control trajectory is defined to be piecewise constant, the state trajectory is approximated using Lagrange polynomials $P_{0:D}$ of degree *D* at Gauss–Radau collocation points $\tau \in R^{D}$ [32]. Hence, the state trajectory is represented by the sum of the weighted polynomials

$$x\left(t_{k+1}\right)\approx C\left(x_{k},c_{k},\tau \right)=\sum_{m=0}^{M}\sum_{n=0}^{N_{x}}c_{k,mn}e_{n}P_{m}\left(\tau \right),\;c_{k,0}=x_{k},\;0>\tau_{0:D}>1,$$

where the polynomial is scaled by the collocation variables $c_{k}\in R^{N_{x}\times D}$ . The additional collocation variables enter the optimization problem over the collocation states $C=\{c_{0}\ldots c_{N-1}\}$ and increase the number of decision variables. To retrieve a physically meaningful state trajectory, the collocation variables require being constrained using the system dynamics. This is enforced by constraining the derivative of the time-scaled polynomial at the collocation points $\tau_{1:D}$ to the ode of the system $\dot{x}\left(t\right)=f\left(x\left(t\right),u\left(t\right)\right)$ evaluated at the same point in time using equality constraints. We obtain *D* equality constraints for each time interval $\left[t_{k},t_{k+1}\right]$ of duration $T=t_{k+1}-t_{k}$ defined by

(10) $$\frac{\partial }{\partial \tau }C_{k}\left(x_{k},c_{k},\tau \right){|}_{\tau_{d}}T^{-1}=f\left(x\left(t_{k}+\tau_{d}T\right),u_{k}\right),\;d=1,\ldots ,D,\;k=0,\ldots ,N-1.$$

The discretization of the state trajectory $X=\{x_{0}\ldots x_{N}\}$ requires further equality constraints to obtain a closed state trajectory. The continuity constraints,

(11) $$Z_{M}x_{k+1}=Z_{M}C\left(x_{k},c_{k},\tau_{D}\right),\;Z_{M}x\left(t\right)=x_{M}\left(t\right),\;\tau_{D}=1.0,\;k=0,\cdots ,N-1,$$

constrain the next state to correspond to the propagated previous state. $Z_{M}\in R^{N_{x}\times N_{x}}$ is defined as a selection matrix for the system model states $x_{M}\left(t\right)$. The continuity constraint is exclusively applied to the system states $x_{M}\left(t\right)$ as defined in Equation (3).

#### 2.4.3. Random Walk

As described in Section 2.3, the biases are modeled by a random walk process. This behavior is introduced by relaxing the continuity constraint

(12) $$\delta_{k+1}\approx \delta_{k},\;k=0,\ldots ,N-1,$$

and defining additional cost terms

(13) $$c_{\delta }\left(x_{k},u_{k},t_{k}\right)=\frac{1}{2}{∥Z_{\delta }x\left(t_{k}\right)-Z_{\delta }x\left(t_{k-1}\right)∥}_{Q_{\delta }^{-1}}^{2},\;k=1,\cdots ,N,$$

where the difference between previous and current state is weighted according to the sensor’s random walk process noise and minimized accordingly. The selection matrix $Z_{\delta }\in R^{3\times N_{x}}$ is used to extract the bias states from the state vector x(t).

### 2.5. MHE Estimator

The components of the optimization problem introduced in Section 2.4 are leading to the definition of the following minimization problem for the MHE estimator

(14a) $$\begin{array}{cc} \underset{X,C,U}{\mathrm{minimize}} & c_{A}\left(x_{j}\right)+\sum_{k=j}^{j+N}\left[c_{p}\left(x_{k},u_{k},t_{k}\right)+c_{a}\left(x_{k},u_{k},t_{k}\right)+c_{\omega }\left(x_{k},u_{k},t_{k}\right)\right]\\ & +\sum_{k=j}^{j+N-1}\left[c_{\delta_{a}}\left(x_{k},u_{k},t_{k}\right)+c_{\delta_{\omega }}\left(x_{k},u_{k},t_{k}\right)\right], \end{array}$$

(14b) $$\begin{array}{c} \text{subject to}\\ \dot{x}\left(t\right)=f(x(t),u(t)), \end{array}$$

(14c) $$Z_{q}x_{j+N}{\left(Z_{q}x_{j+N}\right)}^{T}=1$$

(14d) $$\begin{array}{cc} Z_{M}x_{k+1}=Z_{M}C\left(x_{k},c_{k},\tau \right) & \;k=j,\ldots ,N-1, \end{array}$$

(14e) $$\begin{array}{cc} \frac{\partial }{\partial \tau }C_{k}\left(x_{k},c_{k},\tau \right){|}_{\tau_{d}}T^{-1}=f(x\left(t_{k}+\tau_{d}T\right),u_{k}) & d=1,\cdots ,D,\;k=j,\ldots ,N-1, \end{array}$$

where N∈N and $T=t_{k+1}-t_{k}$ define the horizon length and the sampling time. Since the quaternion is an over-parametrization of a rotation, we have to guarantee that the estimated quaternions satisfy the unit norm condition. The quaternion ode of an orientation as defined in Equation (1c) preserves the unit norm, allowing for adding a single unit norm constraint at the end of an estimation horizon. The constraint (14c) is expressed using the selection matrix $Z_{q}$ for the quaternion entries of the state vector $x_{j+N}$.

The additional cost term in the objective function (14a) defines the arrival cost $c_{A}\left(x_{j}\right),$ which summarizes the history of measurements by penalizing deviations to previous estimates of $x(t_{j})$. The arrival cost is defined as a quadratic approximation of the Schur complement correction and is calculated while marginalizing the system before shifting the horizon [33]. Depending on the nonlinearity of the system, the approximation of the past by the arrival cost might be more or less accurate. In an mhe approach, longer horizons can increase the information about the past by considering more measurement data. The benefit of having a more information about the recent past becomes negligible in cases where the approximation of the past by the arrival cost is already accurate, and vice versa.

## 3. Experimental Results

### 3.1. Dataset

**Figure 3:**
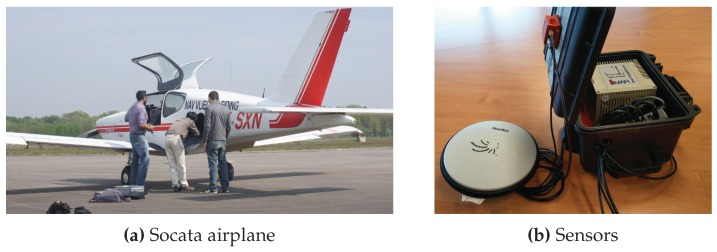
Socata airplane and sensors used for data collection.

**Figure 4:**
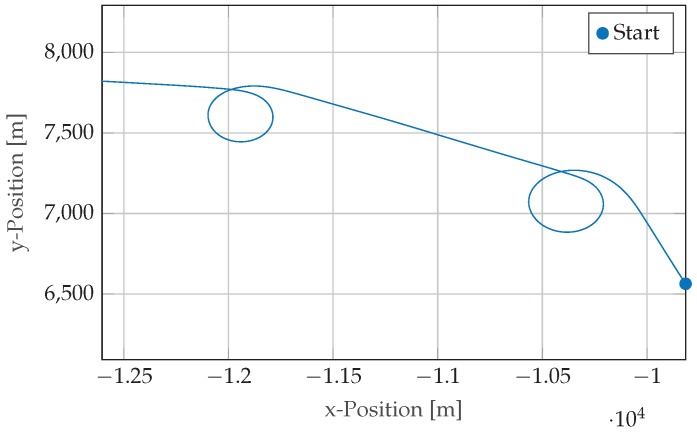
Flight trajectory of airplane used for evaluation (recorded near Enschede, The Netherlands).

The mhe estimator described in Section 2 is used in the following to study the influence of the horizon length *N* on the accuracy of the solution. The dataset used for evaluation has been recorded during a flight with a small Socata single propulsion aircraft (see Figure 3a) flying different maneuvers in hazy weather conditions. In the 100s long data selection, the airplane completes two sharp turns (see Figure 4), while reaching roll angles up to 60deg.

### 3.2. Sensor Setup

The complete sensor setup used for data collection contains two independent sets of sensors: one for the estimation results and one to obtain the reference trajectory. Both sets use a gnss receiver and an imu, which differ in their specifications. While the estimation setup includes exclusively consumer-grade sensors, the reference setup uses higher-grade sensors.

**Figure 5:**
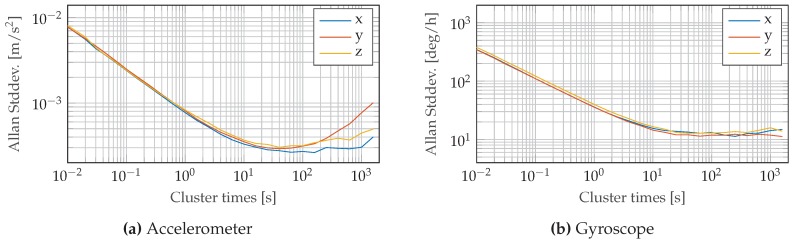
Allan variance curves for accelerometer and gyroscope of the Xsens MTi-700 used for estimation.

To collect the data used for estimation, we used a Xsens MTi-G-700 [34] motion tracker which combines an imu and gnss receiver in a single package. By sharing the same signal pipeline, the risk of time synchronization errors between the independent sensors is reduced. The Allan variance curves of the contained mems accelerometers and mems gyroscopes are plotted in Figure 5 and define the noise density and bias stability of the sensor. The noise density is used to fill the weighting matrices $Q_{\omega }^{-1}$ and $Q_{a}^{-1}$ used in the cost functions (9b) and (9c). The gnss receiver measures the position and its accuracy at time $t_{k}$. The latter is further used to fill the weighting matrix $Q_{p,k}^{-1}$ in Equation (9a). The gnss data ${\bar{y}}_{p,k}^{L}$ was acquired at the maximum rate of 4Hz and the imu was configured to stream motion increments, as described in Section 2.3 at an output frequency of 4Hz. The further available velocity measurements acquired by the gnss receiver are not considered in this document.

Table 1 allows a direct comparison between the noise parameters of the sensors used for estimation and reference. The iMAR-FSAS imu [35] uses a tactical-grade *fibre optic gyro* (fog) with lower measurement noise compared to the consumer-grade mems gyroscopes used for estimation. Furthermore accurate gnss receivers were used in a *differential* gps (dgps) configuration allowing for very precise positioning with errors below 0.1m. To improve the accuracy of the ground truth trajectory further, the acquired data of the reference sensors was fused using a batch processing approach [36].

All sensor devices (Figure 3b) were rigidly attached to the airplane (Figure 3a). The sensor frame *S* of the imu was aligned with the body frame of the aircraft, which implies that no further transformations were required to retrieve physically meaningful output for the navigation states defined in Equation (3).

### 3.3. Algorithm Configuration

Crucial for every kind of nonlinear optimization is the initialization of the decision variables. To initialize the states X and controls U, we first compute the initial orientation $q_{0}^{LS}$ using the velocity vector estimated from consecutive gnss measurements, assuming the system obeys holonomic constraints and has sufficient speed. The measurements of gnss receiver and imu are used to initialize position $p_{0}^{L}$ and controls $u(t_{0})$, respectively. The accelerometer and gyroscope biases are initialized to zero. The resulting initial guess $x(t_{0})$ (as defined in Equation (3)) and its corresponding standard deviations are summarized in Table 2.

### 3.4. Horizon Length Evaluation

To analyze the estimation accuracy depending on the horizon length *N*, the estimation is repeated for different values $N=\left\{1,2,4,8,16,20\right\}$. The horizon length *N* directly defines the number of measurements $N_{M}=N$ and the number of decision variables

(15) $$N_{DV}=N\left(N_{x}+N_{u}+N_{x_{M}}D\right)+N_{x}$$

contained in each mhe optimization problem, where $N_{x}$, $N_{u}$ and $N_{x_{M}}$ represent the dimensions of the state and control vectors defined in Equations (3) and (4). The degree of the collocation polynomials *D* defines implicitly the number of collocation variables used for the integration of the navigation states and is set to D=3 for this evaluation.

**Figure 6:**
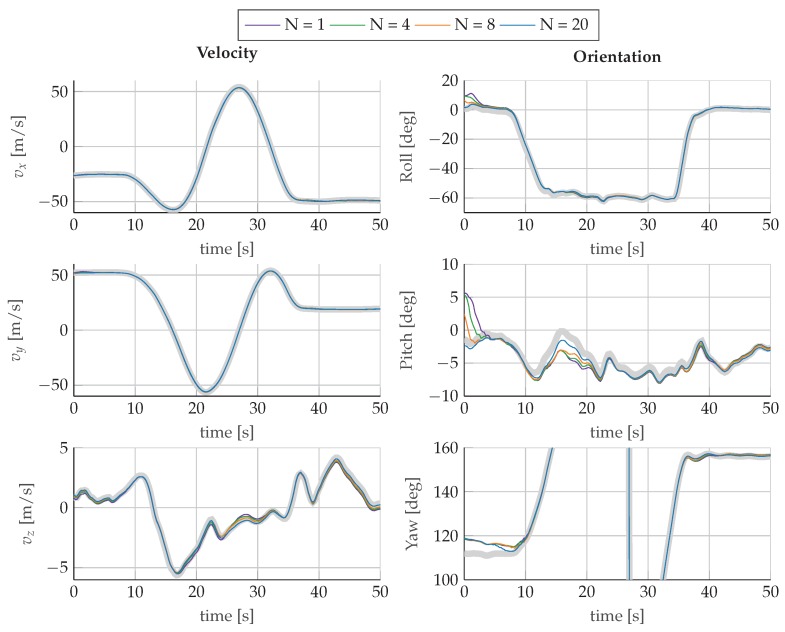
Estimated state trajectories for the first 50 s of velocity and orientation for increasing horizons *N*, compared to the reference trajectory (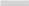).

**Figure 7:**
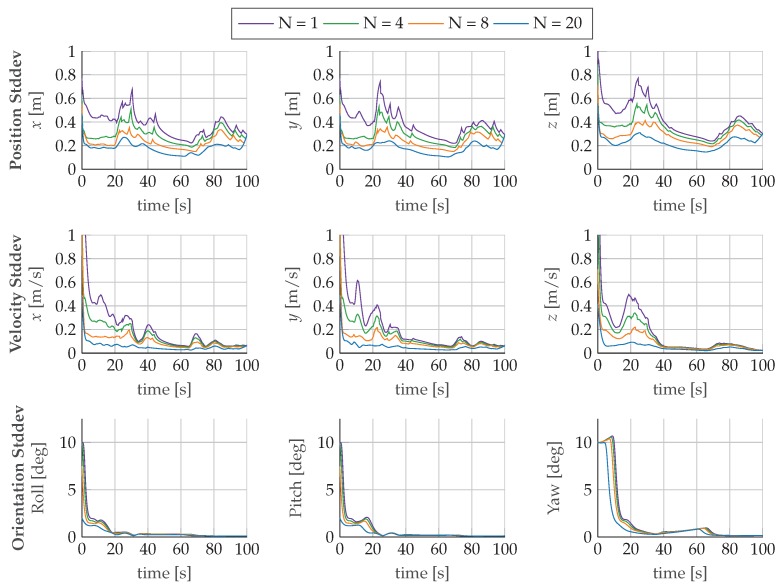
Evolution of standard deviations of the time-lagged estimate of the navigation states: position, velocity, and orientation with increasing horizons *N*.

**Figure 8:**
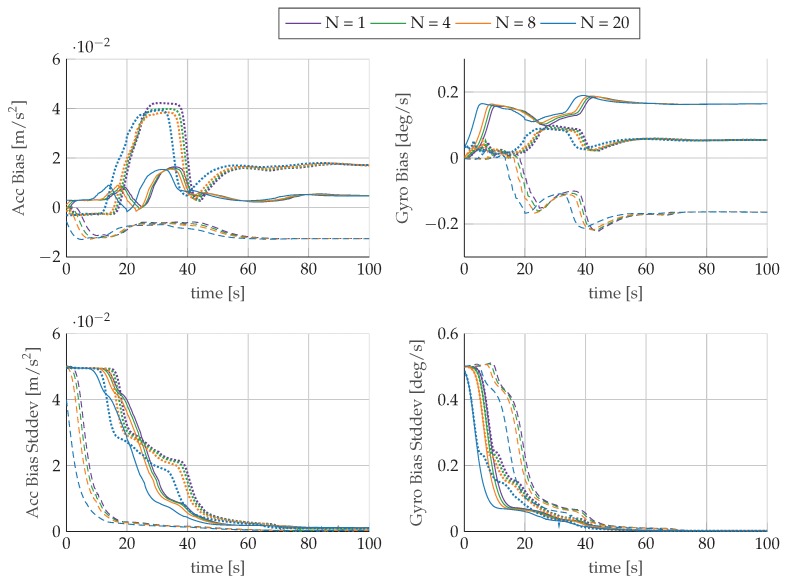
Time-lagged solution for bias values (x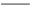, y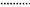, z----) of accelerometer and gyroscope and their corresponding standard deviations for different horizons *N*.

Figure 6 shows the estimated values for velocity and orientation as well as the calculated reference values. For all configurations, the mhe estimator output follows the reference trajectory and no major differences are observable. The plots reveal, however, that the estimation results recover faster from an incorrect initialization with longer horizons and that, in general, larger horizons show an improved tracking behavior of the reference trajectory. The evolution of the standard deviations of position, velocity, and orientation for the evaluated horizons is plotted in Figure 7. The standard deviations converge after the first turn to a steady state, which is determined by the measurement noise. The turn maneuver results in sufficient excitation of the system to observe the orientation, which is the only navigational state that is not directly related to the measurements of the sensors. In Figure 8, we observe that the bias estimates are converging towards the same steady-state for all evaluated horizons. Furthermore, the evolution of the bias standard deviations confirms the necessity of motion changes to identify sensor parameters. The estimated biases converge only after finishing the first turn at around 40s to their corresponding steady state.

Before taking a closer look at the results for different horizons, we define two types of solutions: the real-time solution represents the estimate of the most recent state in the horizon, whereas the lagged solution represents the oldest state in the current window. In the latter case, the value of the state estimate gets updated until it is not contained anymore in the window. Considering a dataset with current data index *j* = 1,..., *J* of total length *J*, the lagged solution can be compared to fixed-lag smoothing, maximizing the probability of *p*(**x**_*j-N*_|**y**_1:*j*_), whereas the real-time solution corresponds to filtering approach estimating *p*(**x**_*j*_|**y**_1:*j*_). For completeness, we remind readers that a typical batch processing approach estimates *p*(**x**_0:*j*_|**y**_1:*J*_). Even though both real-time and lagged solutions contain more measurement data with increasing horizons, there are differences in their interpretation. Both estimators contain an approximation of the past that is defined by the arrival cost *c_A_*(**x***_j_*) in Equation (14a). While the lagged version evaluates the first state in the window, the real-time version estimates the last state in the window, which time is defined by the latest measurement at time *t_j_*. From a time perspective, this means that the lagged version contains information about its past and future, whereas the real-time problem only contains information about its past.

**Figure 9:**
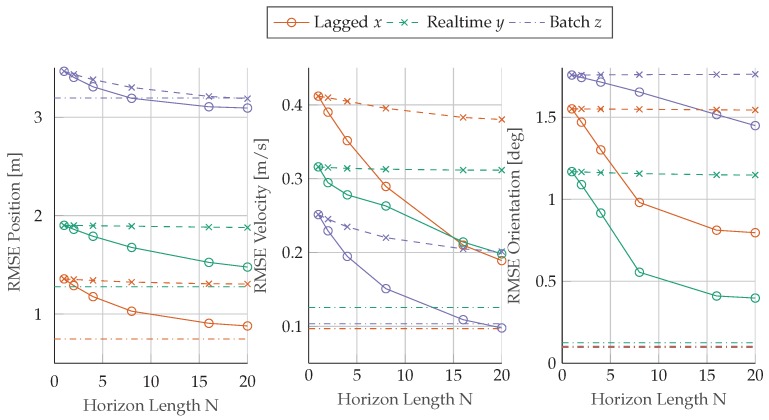
Evaluation of rmses for real-time (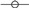) and lagged (−×−) mhe estimators over an increasing horizon length *N* compared to the batch estimation results (−·−·−).

A comparison between real-time and lagged solutions for different horizon lengths is given in Figure 9. A noticeable decrease in terms *root mean square error* (rmse) with increasing horizons can only be observed for the lagged solution. The accuracy of the lagged solution converges towards the solution of the batch estimator (dotted lines), which can be interpreted as a locally optimal solution since all measurement data is contained in the optimization problem, allowing the removal of start-up effects due to the initialization of the algorithm.

In terms of error quantities, we observe relatively large rmses for the position estimate, ranging up to 3.5m in the *z*-direction. The batch solution confirms this large error of 3.1m, which allows the conclusion that the error is not coupled to start-up effects or initialization errors but rather a systematic offset between the sensor setups. Table 1 shows that the gnss receiver, used in the reference setup, operates on the L1/L2-band, which improves position accuracy and especially the altitude estimate compared to a single L1-band gnss receiver as used for estimation.

The orientation estimate reveals error reductions for roll ϕ and pitch θ angles with increasing horizon length. The yaw angle ψ reveals the largest rmse, which is an expected behavior due to limited system dynamics contained in the dataset. The 100s long flight is not subject to larger changes in pitch or strong accelerations in the xy-plane, which contributes to the challenging conditions for the estimation of the yaw angle in the presence of sensor biases. The consequence of a lack of excitation can be further observed in the bias estimates and their standard deviations in Figure 8. The initial jumps of the bias estimates reveal that the biases are not observable under constant motion. After completion of the first turn, the biases in *x*- and *y*-directions become observable and the values converge to their corresponding steady state.

Coming back to Figure 9, we notice that only the lagged mhe solution (solid lines) achieves major rmse improvements in position, velocity, and orientation with an increasing horizon length *N*. The real-time solution does hardly improve by considering a bigger horizon in comparison to the filter solution, where N=1. The evaluated sensor fusion problem exposes a close to linear behavior, which can be approximated well by an arrival cost. Therefore, only small improvements can be achieved by considering a longer window and, therefore, a more detailed representation of the recent past for the real-time solution.

**Figure 10:**
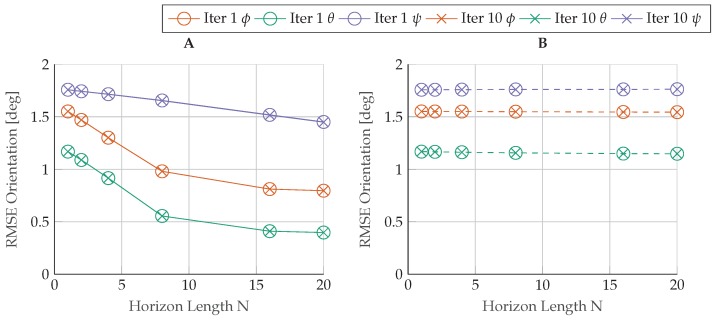
RMSE error in orientation for a different number of iterations I={1,10}. The lagged solutions are plotted on the left (**A**) and the real-time solutions on the right (**B**).

The nearly linear behavior of the regarded problem is further proven by restricting the optimization algorithm to a maximum number of one iteration [37]. This configuration achieves a kf-like algorithm where only a single linearization of the system is calculated. Analyzing the solution in Figure 10 for the rmses in orientation, we notice no visible difference between the single-iteration and the iterated solution that satisfies the exit condition, even though the converged solutions will naturally preserve constraints more accurately.

## 4. Discussion

The presented mhe approach for multi-sensor fusion allows a mathematically elegant formulation in a single optimization problem where cost functions and constraints are recursively adapted with the availability of new measurement data. We introduced the modules of the framework by applying it to the commonly known sensor fusion problem of gnss receiver and imu and evaluated the accuracy of the solution on a real flight dataset in comparison to an accurate reference trajectory for different horizon lengths $N=\left\{1,2,4,8,16,20\right\}$. Major improvements for the navigation states position, velocity, and orientation in terms of rmses can be achieved when considering either the time-lagged or the batch estimates. With an increasing horizon, the rmses decrease and errors approach the results of the post-processed batch solution. For the real-time solution, which estimates the state at the most recent measurement, the performance is only slightly better than the filter solution, corresponding to N=1.

These findings coincide with relevant research [21], where mhe is suggested to improve consistently the accuracy of the estimates at the price of a higher computational cost. In general, it can be said that if applications allow for a time-lagged estimate, the results will be more accurate considering larger horizons. This makes the time-lagged implementation of a mhe-based estimator a versatile approach for systems with slow dynamics and represents an alternative for memory-restrictive offline processing. For real-time critical applications which are governed by nearly linear equations, the accuracy of the state estimates does hardly benefit from the nonlinear optimization approach resulting in a comparable accuracy to filter solutions. The nearly linear behavior of the specific sensor fusion problem was confirmed by evaluating an ekf-like configuration of the mhe estimator using a real-time iteration scheme [37] with a single iteration and a horizon of N=1 yielding similar results in terms of rmses. Notice, the considered sensor fusion problem of imu and gnss receiver can be considered a linear estimation problem when conditioned on orientation, i.e., the optimization problem reduces to linear system if the orientation trajectory is known a priori.

The presented optimization-based framework for sensor fusion allows a seamless and detailed evaluation of filter, mhe, and batch solutions by adapting the horizon length *N*. The modular structure contains several key features which we aim to exploit in further research. By encoding the system dynamics in a single set of odes, the framework can be adjusted to consider more complex system kinematics in a straightforward manner. Additional measurement updates from further sensors can be included by manipulation of the cost function of the optimization problem. When increasing the number of sensors, the calculation of a common time-grid becomes nontrivial. The presented framework is prepared to account for asynchronous measurements by estimating time-continuous state trajectories using a direct collocation approach. The adaptivity of a mhe-based approach for sensor fusion is one major advantage compared to traditional filter schemes and combined with efficient numerical solvers mhe is likely to be beneficial for many challenging applications.

The problem of motion tracking does not classify as nearly-linear problem in the general case. By considering measurements of relative distance sensors, such as *ultra-wideband* (uwb), *time of flight* (tof) or camera sensors, the sensor models become strongly nonlinear. Therefore an mhe approach with the appropriate optimization strategy is expected to improve the real-time estimates as well. For the future, we plan to extend the presented sensor fusion approach of gnss and imu to include the full calibration of the sensors, which will increase the nonlinearity of the optimization problem and mhe is expected to outperform conventional filter techniques. The identification of parameters like scale factors, misalignment or time delays will improve the accuracy when using uncalibrated consumer-grade sensors.

## Figures and Tables

**Table 1:** Sensor specifications for estimation and reference setup.

		Estimation	Reference
**IMU**		MTi-G-700	iMAR-FSAS
Gyro Technology		MEMS	FOG
Gyro Rate Bias Repeatability	[deg/s]	0.2	$0.21\times 10^{-3}$
Gyro Rate Bias Stability	[deg/h]	10	<0.1
Angular Random Walk	$\left[\deg/\sqrt{h}\right]$	0.6	0.16
Accelerometer Technology		MEMS	Servo
Accelerometer Bias Repeatability	[mg]	<5	1.0
Accelerometer Bias Stability	[μg]	40	<10
Accelerometer Random Walk	[μg$/\sqrt{\mathrm{Hz}}$]	80	<50
**GNSS**		MTi-G-700	FlexPak-V2-RT2
Constellation		GPS	GPS + GLONASS
Bands		L1	L1 + L2 (DGPS)
Accuracy	[m]	2–4	0.1
max. Rate	[Hz]	4	40

**Table 2:** Parameters of mhe algorithm defining the initial conditions.

Parameter	Unit	Value	Standard Deviation
Position	[m]	${\bar{y}}_{p,0}^{L}$	10
Velocity	[m/s]	$\frac{d}{dt}{\bar{y}}_{p,0}^{L}$	5
Orientation	[deg]	$q_{0}^{LS}\left(p_{0}^{L},v_{0}^{L}\right)$	8
Accelerometer Bias	$\left[m/s^{2}\right]$	0	0.05
Gyroscope Bias	$\left[\deg/s^{-1}\right]$	0	0.5
